# Early detection of type 2 diabetes risk: limitations of current diagnostic criteria

**DOI:** 10.3389/fendo.2023.1260623

**Published:** 2023-11-09

**Authors:** Jiale Zhang, Zhuoya Zhang, Kaiqi Zhang, Xiaolei Ge, Ranran Sun, Xu Zhai

**Affiliations:** ^1^ Institute of Basic Theory for Chinese Medicine, China Academy of Chinese Medical Sciences, Beijing, China; ^2^ Affiliated Hospital of Nanjing University of Chinese Medicine, Nanjing, China; ^3^ Wangjing Hospital of China Academy of Chinese Medical Sciences, Beijing, China; ^4^ Dongfang Hospital, Beijing University of Chinese Medicine, Beijing, China

**Keywords:** type 2 diabetes, diagnostic criteria, prediabetes, fasting plasma glucose, glycated albumin

## Abstract

Type 2 diabetes (T2D) is the leading cause of diabetes worldwide and is increasing rapidly, especially in youth. It accounts for most diabetes deaths in adults ≥20 years old in the Americas, with type 2 diabetes responsible for most of the disease burden. The incidence and burden of type 2 diabetes in adolescents and young adults have risen in recent decades globally. Countries with lower socioeconomic status had the highest incidence and burden, and females generally had higher mortality and disease burden than males at ages <30 years. Early diagnosis and management are crucial to delaying progression, but current diagnostic criteria based on glucose thresholds and glycated hemoglobin have limitations. Recent analyses show that prediabetes increases cancer risk. Better diagnostic criteria are urgently needed to identify high-risk individuals earlier. This article discusses the limitations of current criteria and explores alternative approaches and future research directions.

## Introduction

Type 2 diabetes (1) is responsible for most diabetes cases globally and is increasing rapidly, particularly in young people. Diabetes accounts for 5.9% of all deaths in adults aged 20 or older in the Americas, with T2D accounting for most of the disease burden (2). The incidence and burden of T2D in adolescents and young adults globally increased from 1990 to 2019. Countries with a low-middle and middle sociodemographic index had the highest incidence and burden rates. Women generally had higher mortality and Disability-Adjusted Life Year rates than men at ages <30 years (3). From 2013 to 2016, 34.5% of American adults had prediabetes (4). According to a 2016 report (5), more than 13 million adults in California (46% of all adults in the state) are estimated to have prediabetes or undiagnosed diabetes. In Chinese adults (6), the estimated prevalence of prediabetes is 35.7%. In September 2008, the Canadian Diabetes Association (7) released diagnostic criteria for prediabetes: fasting plasma glucose (FPG) level of 6.1-6.9 mmol/L or impaired glucose tolerance (IGT) as demonstrated by the oral glucose tolerance test (OGTT). A recent meta-analysis (8) involving 16 studies and 891,426 participants worldwide showed that prediabetes increases cancer risk by 15%. 5-10% of prediabetes patients develop diabetes yearly, and an equal proportion of people recover from high blood sugar levels (9). The global incidence of prediabetes is increasing, and in 2021, the number of people with impaired fasting glucose (IFG) reached 298 million, accounting for 5.8% (10). Experts predict that by 2030, more than 470 million people will have prediabetes (9).

Therefore, early diagnosis and management are crucial for delaying the progression of T2D (11). Generally, HbA1c or patients with blood glucose levels at a subdiabetic level are usually diagnosed with moderate hyperglycemia, known as prediabetes, often called intermediate hyperglycemia. Prediabetes is usually defined as blood glucose concentration above normal but below the diabetes threshold and is a high-risk state for the development of diabetes (12). Although these two concepts may appear similar, they convey distinct meanings. Prediabetes describes the state where blood glucose levels are between normal and diabetic, emphasizing the risk of progressing to diabetes in this stage. On the other hand, moderate hyperglycemia is employed to describe elevated blood glucose levels that are above normal but have not yet reached the threshold for diabetes, placing greater emphasis on the continuum between this stage and diabetes. Therefore, it represents three groups of individuals: those with impaired fasting glucose (IFG), impaired glucose tolerance (IGT), and elevated glycated hemoglobin (HbA1c). The 2023 ADA guidelines (13) still use IFG, IGT, or HbA1c levels of 5.7%-6.4% to diagnose prediabetes. However, current diagnostic criteria may not detect high blood sugar levels until the disease has progressed to a later stage, making it difficult to identify high-risk individuals early. In addition, different organizations and institutions provide different standards, leading to confusion and uncertainty. Recently, a commentary (14) published in The Lancet has brought attention to these issues, underscoring the paramount significance of these aspects. Motivated by previous investigations, we have composed this perspective article. Presently, the conventional standards rely on absolute values of blood glucose thresholds and HbA1c concentrations; however, it is crucial to recognize that high blood sugar status represents a continuous and dynamic process. The existing diagnostic criteria for T2D prove inadequate in early identifying high-risk individuals, leading to delayed diagnoses and heightened health risks (14). Given the limitations of the current diagnostic criteria, we thoroughly examine the insufficiency of these criteria within this article and explore potential alternative modalities and future research focal points.

## Insufficiency of current diagnostic criteria

Current diagnostic criteria for T2D are based on absolute blood glucose thresholds and glycated hemoglobin concentration values, but the hyperglycemic state is a continuous and dynamic process. These limitations may result in misclassification and misdiagnosis, as the American Diabetes Association (ADA) and World Health Organization (WHO) recommend different glucose thresholds and HbA1c levels for diagnosing prediabetes, as shown in **Table 1**. Overall, three main aspects are highlighted.

**Table 1:** Diagnostic criteria for prediabetes.

Index	ADA 2003	ADA 2010	WHO	IEC
Fasting glucose concentration	5.6-6.9mmol/L	5.6–6.9 mmol/L	6.1–6.9 mmol/L	NA
2 h glucose concentration after 75 g glucose load	7.8 – 11.0mmol/L	7.8–11.0 mmol/L	7.8–11.0 mmol/L	NA
HbA1c	NA	5.7–6.4% (39–46 mmol/mol)	NA	6.0–6.4% (42–46 mmol/mol)

ADA, American Diabetes Association; WHO, World Health Organization; IEC, International Expert Committee; NA, Not Available or Not Adopted as Criterion.

First, hyperglycemia is a dynamic and continuum process, meaning that glucose levels are not static but vary with time and other factors. This process can range from normal glucose levels progressively rising to prediabetes and, ultimately, diabetes. Hyperglycemia is not only diabetes but also prediabetes and normoglycemia. 35-50% of prediabetics have a very high risk of developing diabetes within five years (15). In the development of T2D, fasting, and postprandial glycemia become abnormally stable. Postprandial 1-hour hyperglycemia correlates closely with subclinical inflammation and target organ damage, increasing cardiovascular and microvascular complications and death risk. Studies (16, 17) show that 1-hour plasma glucose during OGTT has enhanced predictive capability for T2D, with 1-hour post-OGTT hyperglycemia associated with a 4.27-fold increased risk of retinopathy and a 1.89-fold risk of peripheral vascular complications, plus a 29% higher risk of diabetes mortality. Studies (18, 19) have explored multi-stage models of diabetes progression, with a basic consensus that compensation, stable adaptation, and early decompensation phases typically occur. Initially, there is a long compensatory phase with insulin resistance, increased insulin secretion, and β-cell hypertrophy. In the stable adaptation phase, β-cells cannot fully compensate for increasing insulin resistance, leading to elevated fasting and postprandial glucose levels. The unstable early decompensation phase occurs when β-cells fail to match insulin resistance, causing glucose levels to rise rapidly - typically from prediabetes to manifest diabetes. Research finds (20, 21) that HbA1c criteria can more accurately identify prediabetes as HbA1c reflects average glucose over 2-3 months while fasting plasma glucose (FPG) reflects current levels. Additionally, HbA1c standards avoid FPG limitations, e.g., patients may eat before testing, affecting accuracy. Besides HbA1c and FPG criteria, 2-hour postprandial glucose is used to diagnose prediabetes. However, discrepancies exist between these standards.

There is a lack of consensus on consistent standards and criteria. The ADA and WHO currently provide different glucose thresholds and HbA1c levels for diagnosing prediabetes. The ADA recommends a fasting glucose concentration of 5.6-6.9 mmol/L while WHO defines 6.1-6.9 mmol/L. The ADA also sets a lower HbA1c threshold of 5.7-6.4% (39-46 mmol/mol) for diagnosing prediabetes. There are differences in the diagnostic criteria for prediabetes. Recently, a Chinese study (22) of 2318 subjects found that fasting plasma glucose and HbA1c can diagnose diabetes, but HbA1c should be used cautiously for prediabetes alone. Another Southeast Asian study (23) found significant discordance between fasting plasma glucose and HbA1c measurements in diagnosing diabetes, with fasting plasma glucose underestimating the burden of undiagnosed diabetes. HbA1c-defined diabetes and prediabetes prevalence were 9.7% and 34.6% respectively versus 6.3% and 12.1% for fasting plasma glucose. The weighted kappa statistic for HbA1c concordance with fasting plasma glucose was 0.55, with the most discordance in the prediabetes group. This suggests that using HbA1c as an adjunct test for diabetes diagnosis has significant implications for disease prevalence and clinical practice. Another study (24) found that while HbA1c measurement has advantages like simplicity, standardization and reliability, its performance for screening prediabetes remains debated. Some data show (25) that HbA1c may miss some patients and be influenced by factors like anemia and kidney dysfunction. In contrast, FPG requires fasting and may be affected by medications. Both metrics have pros and cons; the appropriate test depends on the situation. A Japanese study (26) found that using ADA HbA1c criteria of 5.7-6.4% or IFG to assess prediabetes prevalence and progression to diabetes showed that HbA1c screening missed many prediabetics. However, both HbA1c and FPG effectively identified high-risk groups with similar predictions. Thus, combining tests may better target those at risk of developing diabetes for early intervention. Due to ethnic and regional differences, there is no consensus on optimal diagnostic standards. Discrepancies in diagnostic criteria for prediabetes across organizations highlight the need for unified global standards. To achieve this, we propose an international expert committee to systematically review evidence, conduct meta-analyses, and build consensus through discussions. First, convene a diverse, cross-regional panel of specialists to promote unified criteria development. Second, perform comprehensive systematic reviews integrating findings across entities, emphasizing variations in existing criteria and clinical implications. Third, enable regular panel discussions to debate diagnostic criteria merits, align theoretical and practical applications, and share experiences. Fourth, leverage sustained deliberations to reach a consensus and formulate robust, evidence-based unified criteria for population differences. Finally, implement regular evaluations to update criteria per emerging insights, ensuring continued scientific rigor and clinical utility. Through such efforts, consistent standards would optimize prediabetes diagnosis and management globally, thereby improving patient outcomes.

Prediabetes does not necessarily equate to intermediate hyperglycemia. Prediabetes is a continuum with gradually rising glucose levels but does not imply intermediate hyperglycemia. The physiological fasting and 2-hour postprandial glucose concentrations in most prediabetic individuals are typically lower than current diagnostic thresholds so they may go undetected or undiagnosed as prediabetes. Clinically, prediabetics can have normal fasting glucose but 2-hour postprandial glucose between 7.8 and 11.0 mmol/L. HbA1c may be around 6.0%, below diagnostic cutoffs. Thus, prediabetics can potentially reverse diabetes through diet, exercise and medications. Many “prediabetics” have intermediate hyperglycemia, not true prediabetes, so the concept requires a clear definition. Recent papers (11, 14) suggest that a useful intermediate hyperglycemia definition requires clinical relevance, sensitivity and specificity. If thresholds are too high, the definition lacks sensitivity to identify at-risk groups correctly. If too low, it lacks specificity with high false positives incorrectly classifying healthy individuals. Thus, evidence-based definitions of intermediate hyperglycemia are needed from the international community. One study (27) found that the faster fasting glucose levels rise and the higher BMI, blood pressure, triglycerides and lower HDL-cholesterol, the greater the risk of diabetes development. This indicates why more research is needed to determine optimal diagnostic criteria and strategies, and formulate more personalized, precise prevention and treatment plans. However, in addition to existing concepts, we can delve into a pioneering theoretical framework called the pan-glycemia theory. Anchored in pan-vascular diseases, this theory presents a fresh perspective by integrating moderate hyperglycemia and prediabetes. It underscores the significance of a comprehensive and dynamic approach to blood glucose management, aiming to establish a holistic endocrine-vascular health management framework. The pan-glycemia theory emphasizes the entire spectrum of blood glucose management, ranging from the early stages of moderate hyperglycemia to prediabetes. It highlights the importance of dynamic monitoring and personalized interventions. This all-encompassing management approach holds the potential to mitigate the risk of diabetes development while offering more precise strategies for prevention and treatment. Early identification of prediabetes and moderate hyperglycemia through dynamic monitoring and individualized interventions enables timely intervention and treatment. Treatment plans can be customized based on glycemic characteristics and adjusted promptly according to continuous monitoring. Sequential surveillance of blood glucose and other markers gauges treatment response, allowing optimized regimens for better control, lower diabetes risk, and precise management approaches (28). Clinical physicians can develop personalized exercise prescriptions based on patients’ glycemic characteristics and physical conditions. For instance, aerobic exercises such as brisk walking, swimming, or cycling are recommended for insulin-resistant patients to improve peripheral tissue glucose uptake. Endurance training, such as weightlifting or high-intensity interval training can reduce hepatic glucose production for patients with excessive hepatic glucose production. Physicians can improve patients’ glycemic status through individualized exercise prescriptions and provide targeted treatment and prevention strategies.

## Importance of early identification

Type 2 diabetes is a chronic metabolic disease characterized by hyperglycemia. Long-term high blood glucose can damage multiple organs, increasing the risk of cardiovascular disease, kidney disease, retinopathy and other complications (29). Delayed diagnosis of diabetes may increase many health risks. When blood glucose levels remain above normal for an extended period, multiple organ systems can be harmed, raising the risk of cardiovascular, renal, neurological and ocular complications (30). In early diabetes, symptoms may be absent, making it easy to overlook. Untreated high blood sugar can damage blood vessels and the nervous system, leading to various complications. Thus, early identification and treatment of diabetes can effectively reduce these risks. Besides known risk factors, alternative methods can help identify individuals at risk for type 2 diabetes. These methods can improve early identification and intervention of at-risk individuals. The following are three potential alternative diagnostic approaches for prediabetes:

Glycated albumin (GA): GA is a novel biomarker reflecting short-term glycemic variations in diabetic patients (31). Like HbA1c, GA measurement reflects average glucose over 2-3 weeks and can thus monitor and manage diabetes (32). Studies show GA testing has been widely researched and applied (33, 34). It has higher sensitivity and specificity, improving early diabetes diagnosis and treatment (35). The sensitivity of HbA1c plus GA was higher than HbA1c alone (78% vs 50%, P <0.001), detecting nearly 80% of African prediabetics. GA≥17.1% could screen most undiagnosed diabetics while measuring fasting glucose plus GA reduced false-positive screening rates by fasting glucose alone by 33.75% (36). Abnormal GA is an important indicator for OGTT screening in high-risk groups, especially those with normal fasting glucose.

Continuous glucose monitoring (CGM): CGM is a novel technique that continuously records patients’ glucose fluctuations, including postprandial excursions in daily life (37). Compared to traditional glucose testing methods, such as fingerstick measurements or periodic blood tests, CGM offers several advantages. Firstly, CGM provides a complete report of a patient’s glucose profile throughout the day, capturing the dynamic nature of glucose fluctuations. This allows for a better understanding of glycemic variability and identifies specific patterns or trends that may be missed with intermittent testing (38). Moreover, CGM devices can detect glucose changes in response to meals, exercise, stress, and medication, providing valuable insights into how these factors affect blood sugar levels. This information is crucial for patients and healthcare providers in developing personalized treatment plans and making informed decisions regarding diet, physical activity, and medication adjustments. For individuals at risk of developing diabetes, such as those with prediabetes, CGM technology holds potential as a diagnostic and management tool. Participants with a fasting blood glucose level below 100 mg/dL experienced impaired glucose tolerance while continuously monitoring for at least 24 hours (39). CGM identified an additional 15% of prediabetes and 2%, suggesting that abnormal glycemia was more prevalent than before and that the CGM indicator may be more sensitive (40). The 2022 Clinical Practice Guideline for Developing Diabetes Care Plans (41) published by the American Association of Clinical Endocrinologists (AACE) provided a detailed discussion on CGM metrics, application, and data interpretation for assessing glycemic control. Integrating CGM in routine clinical practice improves prediabetes management by providing continuous blood glucose data. Recent advancements in CGM technology (42), such as sensor improvements and device miniaturization, have made CGM systems more reliable and user-friendly. Patients’ increasing demand for better blood glucose control and quality of life has boosted the acceptance of CGM. However, problems exist, such as high costs limiting its use in certain regions and patient populations (43). CGM data requires collaboration and education between healthcare professionals and patients. Technical issues, like sensor drift or data loss, can affect data accuracy. Coverage and reimbursement policies influence CGM’s application, with Medicare supporting it in the US, but CGM is not covered by national insurance in China, and patients bear all costs.

**Figure 1:**
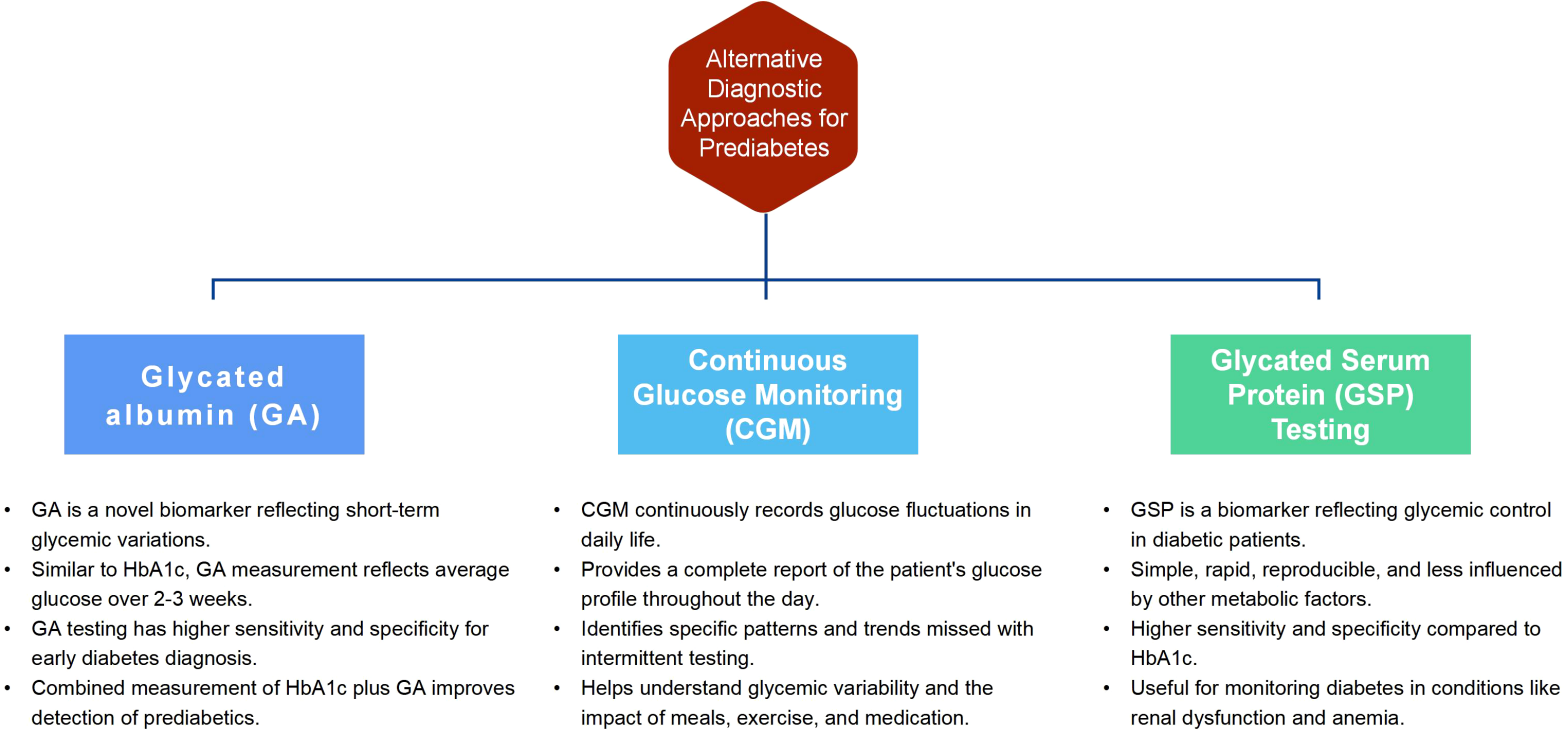
Three alternative placement methods.

Glycated serum protein (GSP) testing: Glycated serum protein (GSP) is a novel biomarker that reflects diabetic patients’ glycemic control (44). GSP detection is simple, rapid, reproducible and less influenced by other metabolic factors. Compared to HbA1c, GSP has higher sensitivity and specificity, making it more accurate for diabetes monitoring in conditions like renal dysfunction and anemia. A study (45) demonstrated a robust positive correlation between fructosamine levels and HbA1c, indicating that fructosamine and HbA1c may serve as useful glycemic biomarkers for patients with concomitant diabetes and cancer, including those undergoing chemotherapy, utilizing glucocorticoids, or with anemia, hypoalbuminemia, or reduced renal function. GSP testing has potential value in early diagnosis of diabetes and diabetic complications (46). A 2016 study (47) found GSP performed well in predicting diabetes based on 2h glucose and HbA1c levels in overweight and obese youth ages 10 to 18. Some research also found that GSP detection has value for screening and predicting diabetic complications (48, 49). In summary, adjunct diagnostic methods may effectively identify individuals at risk for type 2 diabetes. **Figure 1** illustrates the advantages of the three alternative placement methods. **Supplemental Material 1** compares cost-effectiveness, availability, and practicality among the three methods. However, further research evidence is required to support their clinical application in diagnosis. The validation of diagnostic models for prediabetes is crucial to ensure the accuracy and reliability of biomarkers, including GA, CGM, and GSP testing. By validating these biomarkers, their effectiveness in capturing short-term glycemic variations, monitoring glucose levels continuously, and reflecting glycemic control can be confirmed. Establishing the validity of these diagnostic tools enables reliable and accurate prediabetes identification, subsequently facilitating the implementation of appropriate interventions and ultimately improving patient outcomes. In addition, some studies (50, 51) show post-load and 1-hour OGTT glucose levels have higher sensitivity in predicting type 2 diabetes progression than fasting glucose, 2-hour OGTT glucose or HbA1c. Thus,1-hour OGTT glucose may identify high-risk individuals (52). Moving forward, it is critical to identify programs for early identification.

## Conclusion

Given rising type 2 diabetes incidence and complications, identifying and preventing type 2 diabetes early is critical. However, limitations exist in diagnosing prediabetes, with different cutoffs for fasting glucose and HbA1c. Thus, other methods are needed to identify high-risk groups. Prediabetes remains challenging to diagnose due to its obscurity (17). Lack of consensus on prediabetes diagnosis poses challenges in estimating prevalence across populations. Future research should focus on more sensitive, specific biomarkers. Optimizing glucose and HbA1c thresholds and dynamic monitoring are needed to diagnose prediabetes accurately. Exploring optimal strategies to prevent and control prediabetes is important to lower type 2 diabetes risk. In utilizing the new diagnostic model, it is essential to comprehensively explain the decision-making process based on the model’s output. This process enables determining whether an individual belongs to a high-risk group or requires further assessment and management. Several factors influence this decision-making process, including specific patient characteristics, additional clinical considerations, and the incorporation of relevant guidelines or policies. Constructing a comprehensive framework for the decision-making mechanism facilitates a deeper understanding of the potential diagnostic indicators and their practical application in clinical practice. This framework enables healthcare professionals to integrate various factors and considerations, empowering them to make informed decisions and provide tailored interventions and management strategies that address the unique needs of each individual. In summary, international consensus on prediabetes diagnosis, strategies, and more personalized, precise prevention and treatment plans is critical to addressing rising type 2 diabetes incidence. Recognizing hyperglycemia as a continuum, not based solely on glucose/HbA1c cutoffs.

## Data availability statement

The original contributions presented in the study are included in the article/**Supplementary Material**. Further inquiries can be directed to the corresponding authors.

## Author contributions

JZ: Conceptualization, Writing – original draft. ZZ: Formal Analysis, Writing – original draft, Writing – review & editing. KZ: Formal Analysis, Writing – review & editing. XG: Formal Analysis, Writing – review & editing. RS: Conceptualization, Funding acquisition, Writing – review & editing. XZ: Conceptualization, Funding acquisition, Writing – review & editing.
