# Supplementary material for: Early detection of type 2 diabetes risk: limitations of current diagnostic criteria
Source: Front Endocrinol (Lausanne). 2023 Nov 9;14:1260623. doi: 10.3389/fendo.2023.1260623 (PMC10665905; doi:10.3389/fendo.2023.1260623)
Supplement: Supplementary file 1 [file DataSheet_1.docx]

**Supplementary material 1**

Glycated albumin (GA), continuous glucose monitoring (CGM), and glycated serum protein (GSP) are some alternative diabetes diagnostic approaches. Below are some insights into their cost-effectiveness, availability, and utility across diverse healthcare contexts:

**Glycated albumin (GA):**

Cost-effectiveness: GA is typically less expensive than HbA1c tests. However, the cost can add up with frequent monitoring. More studies on long-term cost-effectiveness, taking into account glycemic control are warranted.

Availability: GA is widely available in clinical labs, unlike fructosamine. However, the availability of standardized GA assays is still limited in some regions, affecting clinical utility.

Utility: GA provides short-term glucose control data complementing HbA1c. However, evidence is still insufficient if GA can replace HbA1c in diagnosis or management guidelines. More studies confirming its utility are needed.

**Continuous glucose monitoring (CGM):**

Cost-effectiveness: Upfront costs of CGM are high but may lead to long-term savings from reduced complications. Cost-effectiveness depends on reimbursement policies and eligibility criteria.

Availability: CGM access is increasing globally but remains restricted due to high costs. Strategies like shared or intermittent use of CGM in low-resource settings need evaluation.

Utility: CGM enables personalized care but requires comprehensive patient education for optimal utility. Evidence on whether CGM improves outcomes compared to self-monitoring blood glucose is still inconsistent.

**Glycated Serum Protein (GSP):**

Cost-effectiveness: Compared to HbA1c, GSP may have a higher cost due to being a newer marker with limited prevalence. However, studies have shown that GSP can more accurately assess blood glucose control in patients with type 2 diabetes, suggesting potential long-term cost-effectiveness. GSP reimbursement policies vary across countries and regions, impacting its cost-effectiveness in other healthcare systems.

Availability: The detection method for GSP is relatively complex, primarily utilizing mass spectrometry techniques. This limitation restricts its application in primary healthcare institutions. Currently, GSP testing is mainly concentrated in university hospitals and research institutions. With technological advancements, future detection methods for GSP may become more simplified, enhancing its availability in primary care settings.

Utility: GSP can detect glycated proteins with lower molecular weight, allowing for the assessment of shorter-term blood glucose control and increased sensitivity to glucose fluctuations. It is not influenced by red blood cell lifespan or certain types of hemoglobin variability. GSP can provide additional information on blood glucose control and assess medication effectiveness in clinical settings. However, the advantages of GSP in guiding clinical treatment require further confirmation through large-sample studies.

**Summarize**

Cost-effectiveness: Continuous Glucose Monitoring (CGM) is generally considered a more expensive option, including purchasing and maintaining the devices and sensors. Glycated Albumin (GA) and Glycated Serum Protein (GSP) may be relatively more affordable, but specific costs can vary depending on laboratory testing methods and regions.

Availability: CGM and GA are typically feasible in most healthcare settings as they can be analyzed using standard laboratory equipment. However, the availability of CGM may be limited by factors such as device supply and training. As for GSP, there are fewer commercially available testing methods, and it may be conducted only in specific laboratories or research institutions.

Utility: CGM provides continuous glucose data, monitoring glucose fluctuations and adjusting treatment plans. GA and GSP can provide information on recent blood glucose control, which may help evaluate treatment effectiveness and for patients whose HbA1c accuracy may be affected in specific situations.
